# Elucidating the Effect of Accelerated Carbonation on Porosity and Mechanical Properties of Hydrated Portland Cement Paste Using X-Ray Tomography and Advanced Micromechanical Testing

**DOI:** 10.3390/mi11050471

**Published:** 2020-04-29

**Authors:** Hongzhi Zhang, Claudia Romero Rodriguez, Hua Dong, Yidong Gan, Erik Schlangen, Branko Šavija

**Affiliations:** 1 Microlab, Faculty of Civil Engineering and Geosciences, Delft University of Technology, 2628 CN Delft, The Netherlands; hzzhang@sdu.edu.cn (H.Z.); H.Dong@tudelft.nl (H.D.); y.gan@tudelft.nl (Y.G.); Erik.Schlangen@tudelft.nl (E.S.); B.Savija@tudelft.nl (B.Š.); 2 School of Qilu Transportation, Shandong University, Jinan 250002, China

**Keywords:** hydrated cement paste, carbonation, micromechanical properties, porosity

## Abstract

Carbonation of hydrated cement paste (HCP) causes numerous chemo–mechanical changes in the microstructure, e.g., porosity, strength, elastic modulus, and permeability, which have a significant influence on the durability of concrete structures. Due to its complexity, much is still not understood about the process of carbonation of HCP. The current study aims to reveal the changes in porosity and micromechanical properties caused by carbonation using micro-beam specimens with a cross-section of 500 μm × 500 μm. X-ray computed tomography and micro-beam bending tests were performed on both noncarbonated and carbonated HCP micro-beams for porosity characterization and micromechanical property measurements, respectively. The experimental results show that the carbonation decreases the total porosity and increases micromechanical properties of the HCP micro-beams under the accelerated carbonation. The correlation study revealed that both the flexural strength and elastic modulus increase linearly with decreasing porosity.

## 1. Introduction

Carbonation is a well-known spontaneous process commonly occurring in hydrated cement paste. It is related to hardening, durability, and aging mechanisms of cement-based materials [1]. In this context, carbonation is a chemical reaction in which hydration products (e.g., calcium hydroxide Ca(OH)_2_, calcium silicate hydrate (C-S-H), and calcium aluminate hydrate (CAH)) react with atmospheric carbon dioxide in the presence of moisture to form calcium carbonate (CaCO_3_) [2].

Carbonation can be classified as passive or active [3,4]. Passive carbonation (also termed natural carbonation) usually occurs on the hardened concrete cover. It reduces the pH of the pore solution from 12.6 to values lower than 9 (as low as 8.3 [5]). In reinforced concrete, this may destroy the protective oxide film on the steel surface, initiate reinforcement corrosion, and cause cracking of the concrete cover due to rust buildup [6,7,8]. Therefore, carbonation is commonly considered to be one of the most deleterious deterioration mechanisms of reinforced concrete. However, controlled formation of calcium carbonates in the pore structure of unsaturated concrete could be considered as a positive aspect in Portland cement concrete, since it may increase its mechanical strength [9,10].

Active carbonation usually occurs under an accelerated controlled environment [11]. There has been a number of recent advances aiming at utilizing the ability of cement paste to react with carbon dioxide to achieve certain economical or environmental benefits (or both). With a relatively high concentration (>99%) of CO_2_ condition, the reaction between CO_2_ and calcium-bearing phases of fresh concrete can occur within a few minutes to shorten the time of curing [12,13,14]. Through active carbonation, the properties of concrete can be improved by gaining a higher early strength and surface hardness. This can be considered an active decision to take advantage of this reactivity. Alternatively, active carbonation can be applied to improve the quality of recycled concrete aggregate consisting of a porous hydrated cement paste outer layer [15,16,17]. The formation of CaCO_3_ can reduce the porosity and densify the porous surface, thus reducing the amount of additional water required for the mixing [18]. This can significantly enhance the strength and durability of recycled concrete [19].

To provide the basis for active use of carbonation as a tool for manipulating certain properties of cementitious materials, numerous efforts have been devoted to fundamentally understanding the influence of carbonation on the microstructure–properties relationship. However, when laboratory sized samples (centimeter range in general) are used, homogeneous carbonation can hardly be ensured, and the existence of carbonation-associated cracks is often observed. Therefore, these tests cannot be used to reveal the microstructure–mechanical properties relationship. In some studies [20,21,22], nanoindentation has been used to show local changes in hardness and elastic modulus values due to the carbonation of various binders. However, parameters such as strength cannot be directly assessed by this technique. For this purpose, newly developed universal small-scale testing approaches [23,24,25,26,27] can be used. In such approaches, micro-scale sized specimens are produced using a micro-dicing saw. These micro-scale sized specimens also provide the possibility to achieve homogeneously carbonated and uncracked samples.

In this study, micro-beam specimens with a cross-section of 500 μm × 500 μm were prepared and subjected to an accelerated carbonation treatment under unsaturated conditions. The changes of the mechanical properties were measured by a miniaturized three-point bending test, and changes of the total porosity were obtained by dual micro X-ray tomography. The influence of porosity changes on the micromechanical properties was studied based on the experimental results.

## 2. Materials and Methods

### 2.1. Materials

Specimens of cement paste with two water to cement ratio w/c ratios (0.3, 0.4) were prepared by mixing ordinary Portland cement (CEM I 42.5 N, ENCI, Maastricht, Netherlands) and demineralized water in a Hobart planetary mixer. The pastes were mixed in accordance with EN 196-3:2005+A1:2008 (E). First, cement was mixed with water for 90 s at speed 1. The mixer was then stopped for 30 s during which all paste adhering to the wall and the bottom part of the bowl was scrapped using a metal scraper and added to the mix. The mixing was then resumed for an additional 90 s. The total mixer running time was around 3 min. After mixing, pastes were cast in plastic cylinders with a 24-mm diameter and 39-mm height. Fresh mixtures were compacted on a vibrating table to remove air bubbles. The cylinders were sealed with paraffin film and a lid at the top to prevent any exchange of moisture or CO_2_ with the environment. In order to prevent bleeding, sealed cylinders were rotated slowly (2.5 rpm) for 24 h. Afterwards, the specimens were cured under sealed conditions in a laboratory environment (approximately 20 °C) for one year until testing.

### 2.2. Sampling

**Figure 1:**
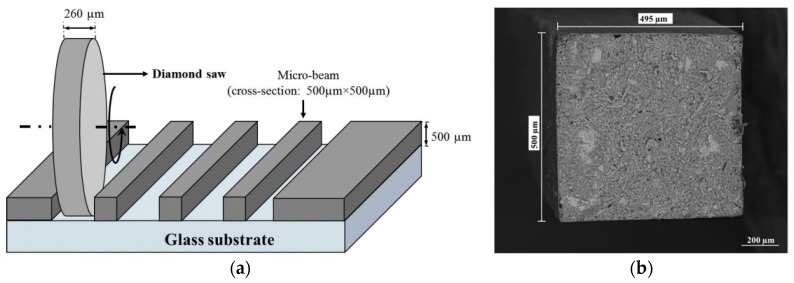
(**a**) Schematic view of sample preparation. (**b**) ESEM image of the cross-section of the micro-beam [24].

After the curing period, the cylindrical specimens were sliced transversally and the top and bottom of the cylinders were discarded to prevent the use of carbonated material. The core slices were used for preparation of micro-beam specimens, with a cross-section of 500 μm × 500 μm and lengths varying between 1 and 3 cm, for the X-ray computed tomography and micro-beam bending tests. For the micro-beam preparation, a “Struers SystemAbele Accessories” apparatus was first used for grinding and polishing of the paste specimens to reduce the thickness of the cement paste to a thickness of 500 μm. In this process, the slice was mounted on a glass substrate using a UV glue and then ground down to 1 mm using diamond ring grinding discs with a grit size of 125 μm to 30 μm in descending order. Afterwards, the glue was dissolved with acetone. The slice was glued on another glass substrate from the other side. Further grinding was then performed until the required thickness was achieved. The second step consisted of sawing the micro-beam specimens from the prepared thin slice. A micro-dicing saw ‘‘MicroAce Series 3” (Loadpoint Ltd., Swindon, UK) was used on the thin section for this purpose. Dicing was performed as schematically shown in Figure 1. The obtained micro-beam specimens were then unglued using acetone. Note that the specimens were stored in acetone prior to the carbonation exposure for at least 24 h. Some of the specimens were used for investigations of porosity and mechanical properties as the reference, i.e., uncarbonated conditions. The remaining specimens were subjected to accelerated carbonation in a carbonation chamber set at laboratory temperature (20 °C) and 65% Relative Humidity (RH) and with a CO_2_ concentration of 3%, without further conditioning. Exposure to CO_2_ was allowed from all the surfaces of the micro-beams. Dummy samples of the same cement pastes were also put in the carbonation chamber to monitor the carbonation depth through phenolphthalein spraying and measurement of the depth with a caliper. When the carbonation depth of the dummy specimens was greater than 250 μm, the micro-beams were deemed to be completely carbonated and their total porosity and bending behaviour were assessed.

### 2.3. Micro-Beam Bending Test

**Figure 2:**
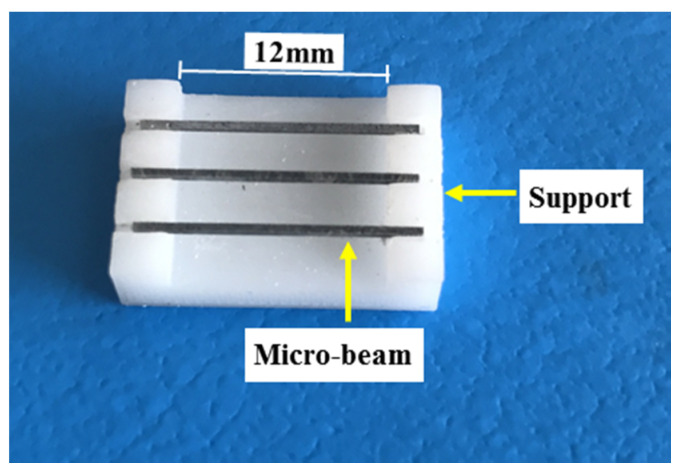
Micro-beam specimens placed on a 3D-printed support, after [24].

The three-point bending of the micro-beam test [23] previously developed by the authors was employed to determine the micromechanical properties (i.e., elastic modulus and flexure strength) of cement paste before and after carbonation. In this test, the micro-beam specimens (obtained as shown in Figure 1b) were subjected to three-point bending. This test was performed in the nanoindenter using a cylindrical wedge tip (radius 9.6 μm, length 700 μm). The micro-beams were placed on a 3D-printed plastic support with a span of 12 mm (see Figure 2). This length is much longer than the length of the cross-section to minimize the influence of shear on the measured flexural strength. The resulting aspect ratio of the micro-beam was 28. A line load was applied in the middle-span of the beam through the tip. Experiments were run under displacement control with a rate of 500 nm/s. The load and displacement responses were recorded by the indenter automatically.

**Figure 3:**
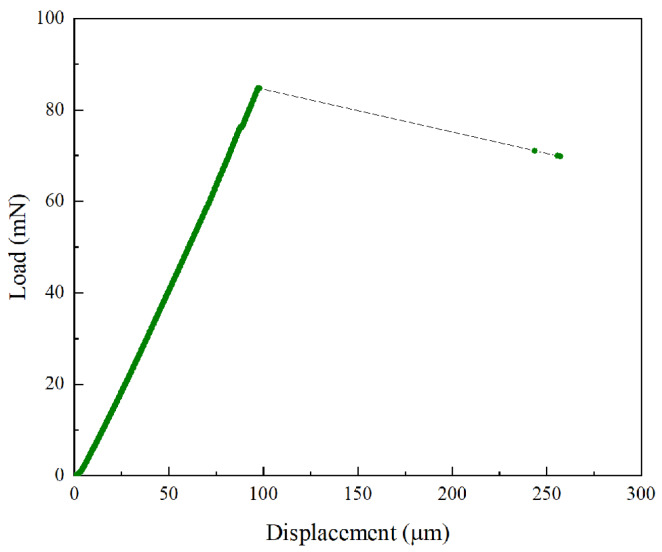
A typical load vs. displacement curve measured in the miniaturized three-point bending test.

Figure 3 shows the typical recorded load-displacement diagram in which the maximum force *F*_max_ was used for the flexural strength calculation using elastic beam theory [28]:

(1) $$f_{t}=\frac{3F_{\max}L}{2d^{3}},$$

where *L* is the length of the span and d is the cross-sectional dimension of the square. In terms of the calculation of Young’s modulus (*E*), data of recorded load displacements in the range between 50% and 80% of maximum load were used, as plots in this range are approximately linear to the point of fracture. Young’s modulus E was evaluated as [28]:

(2) $$E=\frac{L^{3}}{4d^{4}}S,$$

where *S* is the average slope in the range between 50% and 80% of the maximum load.

### 2.4. Micro X-ray Computed Tomography

**Figure 4:**
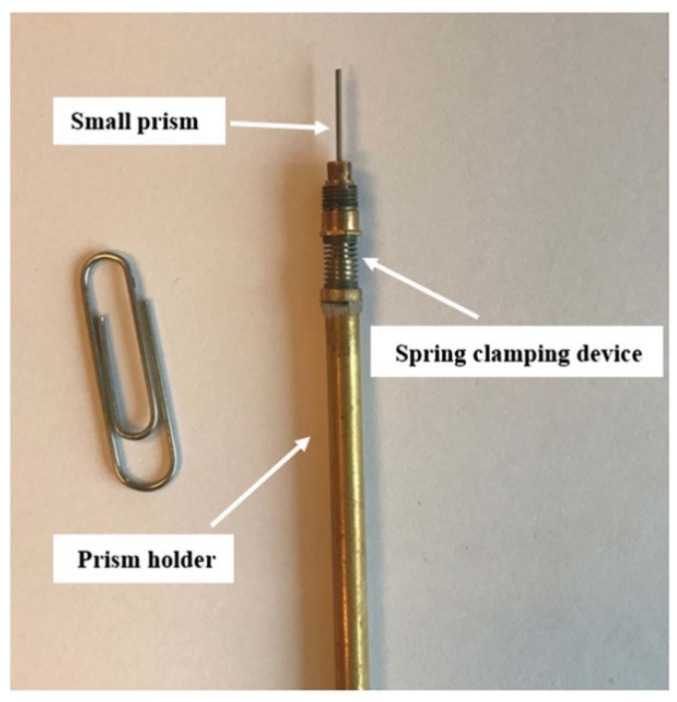
A micro-beam clamped on a custom-made holder, prior to X-ray CT scanning [29].

To quantify the total porosity of noncarbonated and carbonated cement paste, the micro-beams were scanned using a Micro X-ray Computed Tomography (XCT) Scanner (Phoenix Nanotom, Boston, MA, USA). The X-ray tube was operated at 125 kV and 60 μA with a spot size of 500 nm. In total, 1800 projections were acquired by a digital GE DXR detector (2288 × 2304 pixels). Each projection was the average of four radiographs with an exposure of 500 ms to minimize noise. Owing to the small dimensions of the sample and the need for close proximity to the source (7.5 mm), the sample was clipped with an in-house device, as can be seen in Figure 4. The voxel resolution under these conditions was 750 nm.

After calibration of the projections with dark and bright field images, the 3D reconstruction of the acquired projections was carried out with the XCT scanner proprietary software, Phoenix datos|x 2.0. Ring and beam hardening artifacts were corrected during the reconstruction with built-in algorithms. Further image analysis was carried out in three representative elementary volumes (REV) for each beam. The REVs consisted of volumes of 500 × 500 × 500 voxels (350 × 350 × 350 μm).

#### 2.4.1. Microstructure Segmentation

**Figure 5:**
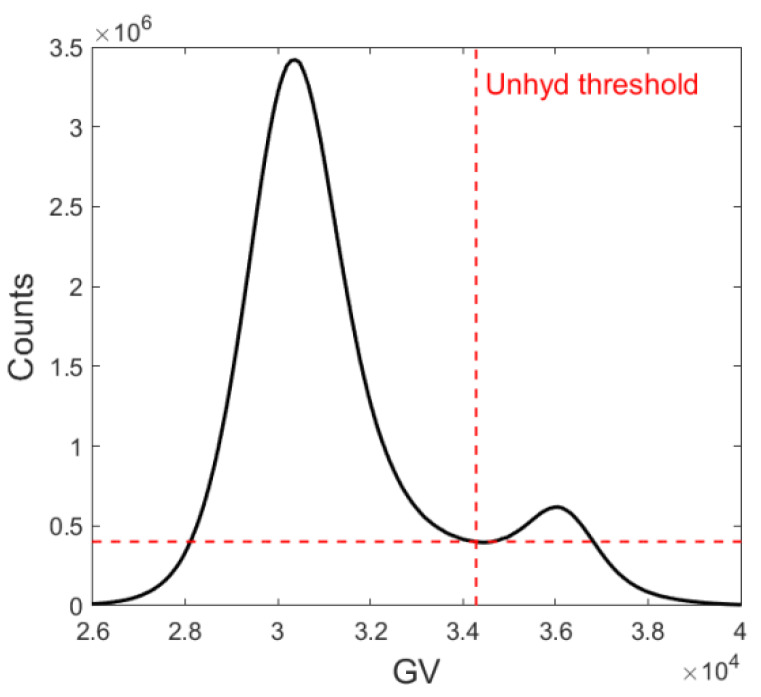
XCT histogram in which a well-defined Gaussian bell of unreacted cement grains occurs.

The attenuation contrast between hydrates and anhydrous cement grains was reflected in the gray value histograms of the resulting reconstructed volumes. The unreacted cement grains were denser than their derived reaction products, thus having a well-defined Gaussian bell in the XCT histograms, see Figure 5. The threshold was chosen arbitrarily as the grayscale value (GV) at the minimum of the valley between the two Gaussian bells [23]. The degree of hydration (α) was estimated according to Powers & Brownyard’s assumption [30] that the volumetric fraction of hydration products (*F*_hp_) is 2.1 times that of their original anhydrous grains (*F*_ag_) through:

(3) $$\alpha =\frac{\frac{F_{\mathrm{hp}}}{2.1}}{\frac{F_{\mathrm{hp}}}{2.1}+F_{\mathrm{ag}}},$$

#### 2.4.2. Total Porosity Characterized Through Dual CT

Dual micro X-ray tomography was used in this study to quantify total porosity. Herein, total porosity refers to the porosity of cement paste that can be accessed by water. This method, as described in [31,32], resolves the total porosity of cement paste per voxel by using the linear rule of additivity of the attenuation coefficients weighed with the volumetric fractions of cement paste, pores, and any fluids in them. For each specimen, two scans are performed on the porous cement paste: one in the dry state and one after saturation with a doping solution of potassium iodide (KI) with molarity of 0.05 M. If scanning settings and pore structure are kept constant for both scans (i.e., unsaturated and saturated), a linear proportion can be established between the attenuation coefficient of the voxel and its GV obtained after reconstruction. In order to account for unavoidable changes in beam intensity between the two scans, the scans are calibrated by using the GV of air and anhydrous grains which remain theoretically unvaried after saturation. The voxel total porosity, *ϕ*(x), can then be calculated as:

(4) $$\phi (x)=\frac{{\mathrm{GV}}_{\mathrm{sat}}-{\mathrm{GV}}_{0}}{{\mathrm{GV}}_{\mathrm{KI},\mathrm{sol}}-{\mathrm{GV}}_{\mathrm{air}}},$$

where GV_0_ and GV_sat_ are the calibrated greyscale values of the voxel x before and after vacuum saturation with KI solution, respectively. GV_KI,sol_ and GV_air_ are the calibrated greyscale values of the KI solution bath and of the air surrounding the sample. 

For the scan performed in the dry state, drying of the sample was done through solvent exchange with acetone, followed by approximately 24 h in a glove box in a nitrogen atmosphere (for noncarbonated samples) or in a desiccator (for carbonated samples). In the case of the scan performed in the saturated state, the samples underwent vacuum saturation with KI solution for approximately 24 h. In order to avoid drying during the scan, the sample was kept submerged throughout.

To perform the voxel-wise operation described in Equation (4), the two resulting volumes were registered through the open source software DataViewer from Bruker (Billerica, MA, USA). In this way, it was possible to establish spatial correspondence of each voxel for both scans. A median filter with radius of 2 pixels was performed on the reconstructed volumes to prevent noise propagation. Image analysis was then carried out through the freeware ImageJ.

## 3. Results and Discussion

### 3.1. Micromechanical Properties

**Figure 6:**
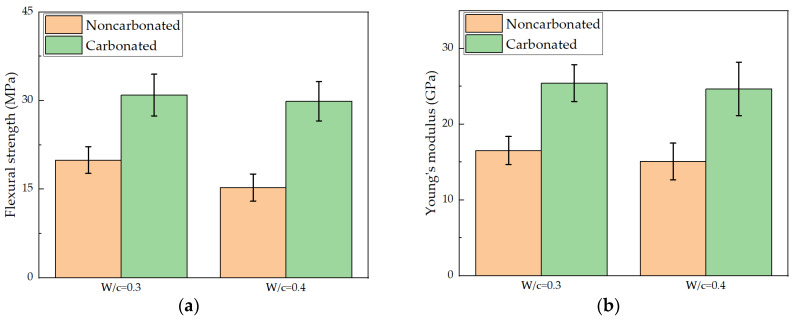
Measured mechanical properties of hydrated cement paste micro-beams: (**a**) flexural strength; (**b**) Young’s modulus.

For each condition, 20 micro-beams were tested. The average and standard deviation of the measured flexural strengths and Young’s moduli for carbonated and noncarbonated hydrated cement pastes are shown in Figure 6. As expected, flexural strengths of the micro-beams are more than one order of magnitude higher than the macroscopic values of cement paste or concrete in the meso-scale (typically a few MPa [1,33,34]). This is in accordance with the size effect of quasi-brittle material in which theoretical strength increases as the sample size or scale decreases [35,36,37].

Even more important, however, is the increase in both flexural strength and Young’s modulus that were observed after the carbonation. This trend has also been observed at the meso-scale [38,39,40]. However, more significant increase was observed in the current study. This is because in the laboratory sized specimens, only the surface is strengthened by the carbonation process, while the micro-beams are fully carbonated. Furthermore, differential carbonation shrinkage may occur in the meso-scale specimens, which may cause micro-cracks in the specimen leading to a decrease of mechanical properties [41,42,43]. In terms of the flexural strength, the mean value increases from 19.93 MPa to 30.93 MPa for a w/c ratio of 0.3 and from 15.28 MPa to 29.87 MPa for a w/c ratio of 0.4. After carbonation, the two hydrated cement pastes almost reach the same flexural strength. A similar trend was observed for Young’s modulus as the Young’s moduli all increased to around 25 GPa (25.43 for w/c = 0.3 and 24.67 for w/c = 0.4) after carbonation. This is related to the decrease of porosity as described later and possibly also structural changes of the C-S-H gel which is the main binding component in cement [33]. As reported by Han et al. [20], the nanoindentation test shows that the hardness and elastic modulus tend to shift towards higher values in Portland cement paste after carbonation. However, this might not be the case for low-pH HCP, such as that incorporating supplementary cementitious materials, as less portlandite is available for carbonation, which changes the kinetics and nature of the process [44,45]. Additionally, a greater increase in micromechanical properties is therefore observed for w/c ratio 0.4.

### 3.2. Assessment of Carbonation Homogeneity

**Figure 7:**
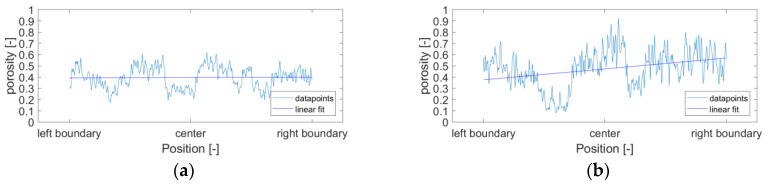
Porosity profiles along two cross-sections of carbonated micro-beams for paste with w/c = 0.3 (**a**) and w/c = 0.4 (**b**).

As a way to further assess the homogeneity of the carbonation degradation within the micro-beams, porosity profiles, across the central region of the micro-beams, are shown in Figure 7 a,b) for carbonated HCP with initial w/c = 0.3 and w/c = 0.4, respectively. The porosity profiles were obtained by averaging the GV difference (numerator of Equation (4)) along the central region thickness (80 μm) for each pixel position along the section and normalizing to the GV difference between the GV of the KI solution and air (denominator of Equation (4)). To mitigate the effects of degree of hydration on the porosity profiles, null differences were not considered for the averaging. Nevertheless, localized drops in porosity in the profiles were observed to be in correspondence with the presence of anhydrous grains, for which GV does not change after saturation with KI solution. The latter may be an indication that porosity values around the anhydrous grains are lower due to the higher density of the surrounding C-S-H gel.

As observed from the profiles, the voxel porosity oscillates along the profile but no clear increase in the porosity is present at the centre of the profiles. The latter finding indicates that the distribution of porosity values per voxel is not correlated with the position and therefore, carbonation of the samples can be considered homogeneous. It is also possible to observe that porosity values for paste with w/c = 0.4 have a higher scatter than the ones from HCP with w/c = 0.3.

### 3.3. Degree of Hydration and Total Porosity from Micro CT

**Figure 8:**
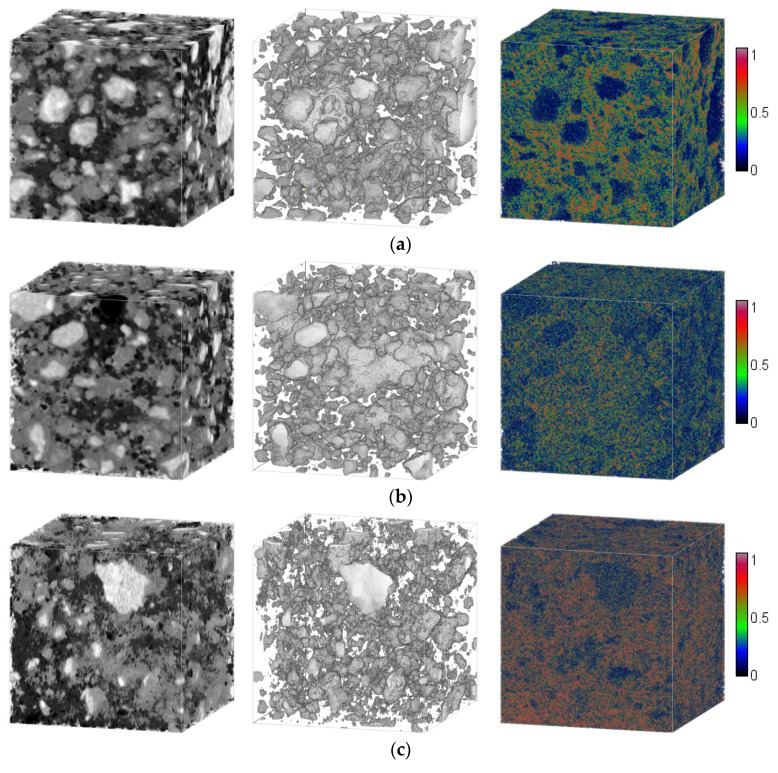

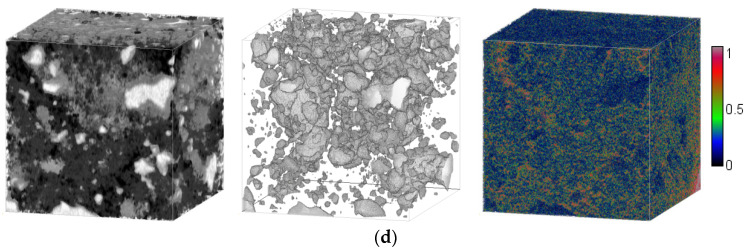
Examples of GV REV (**left**), segmented anhydrous grains (**middle**) and voxel total porosity distribution (**right**) for (**a**) noncarbonated cement paste with w/c ratio of 0.3; (**b**) carbonated cement paste with w/c ratio of 0.3; (**c**) noncarbonated cement paste with w/c ratio of 0.4; (**d**) carbonated cement pastes with w/c ratio of 0.4.

**Figure 9:**
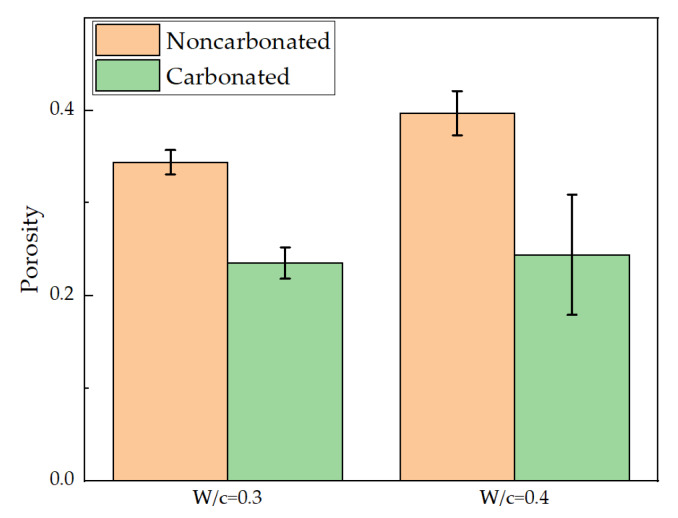
Total porosity of studied pastes before and after carbonation, characterized through dual CT.

Figure 8 shows examples of a greyscale value REV and respective segmented anhydrous grains and voxel total porosity distribution for each of the studied cement pastes, noncarbonated and carbonated. The obtained values of total porosity are summarized in the bar graph in Figure 9 for the studied pastes, before and after accelerated carbonation.

From the segmentations of anhydrous phases, the presence of larger, closely spaced cement grains in the paste with w/c ratio of 0.3 is evident. The paste with a w/c ratio of 0.4 instead shows smaller anhydrous grains. The degrees of hydration for the pristine pastes were 0.784 ± 0.026 and 0.919 ± 0.015 for water-to-cement ratios of 0.3 and 0.4, respectively. Such values agree very well with the results obtained by Bentz [46] via thermogravimetry for similar pastes sealed for one year. In terms of the carbonated pastes, the degrees of hydration were estimated as 0.773 ± 0.014 and 0.875 ± 0.031, for w/c ratios 0.3 and 0.4, respectively. The values of α before and after carbonation, corresponding to paste with w/c = 0.3, were very close. On the other hand, for HCP with w/c = 0.4, the average degree of hydration after weathering was 5% lower than before. A possible cause is that calcium carbonate density (2700 kg/m^3^) is close to that of the anhydrous cement grains (2900–3150 kg/m^3^) which may have increased the amplitude of the valley between the Gaussian bells of anhydrous grains and hydrates. Because of the choice of the threshold at the minimum of that valley, the latter effect may have had the consequence of overestimating the number of voxels segmented as unreacted cement. For the cement paste with a w/c ratio of 0.3, with a lower degree of hydration and coarser anhydrous grains, this increase in volume of unhydrates was less significant than for a w/c ratio of 0.4. In any case, the degrees of hydration did not increase for the pastes during carbonation which indicates that no further hydration occurred during the weathering regime and that the investigated changes in total porosity were due to the sole carbonation mechanism.

Using the model proposed in [47] and the degrees of hydration obtained from the CT data, it was possible to obtain the hydrates’ theoretical volumetric fractions for 1-year-old pastes with w/c ratios 0.3 and 0.4. The results are summarized in Table 1. The volumetric fraction did not differ significantly between the studied pastes.

As already reported in the literature [48,49], lower water-to-cement ratios in pristine pastes resulted in higher values of percolated porosity. In the case of paste with w/c = 0.3, the total porosity measured was 0.344 ± 0.013 whereas for w/c = 0.4 the value was 0.397 ± 0.024. As expected, such values were higher than those obtained by techniques such as MIP [50,51] due to the documented drawbacks of the method i.e., ink-bottle effect. The measured values in this study agree well with other results found through methods that rely on the absorption of water, such as gravimetry and sorption isotherms. For example, Baroghel-Bouny [52] compared the porosity obtained by gravimetry and sorption isotherms of OPC pastes with water-to-cement ratios 0.35 and 0.45 cured for two years under sealed conditions. The porosity measured through sorption isotherms was approximately 0.31 for w/c = 0.35 and 0.41 for w/c = 0.45. The gravimetric results yielded values of total porosity of 0.32 and 0.44, for w/c = 0.35 and 0.45, respectively.

After the accelerated carbonation regime, the total porosity of both cement pastes decreased considerably. The total porosity of carbonated paste with w/c = 0.3 was 0.235 ± 0.017, 30% lower than in the noncarbonated state. In the case of paste with an initial w/c ratio of 0.4, the decrease in total porosity was 40%. The final value of total porosity for the latter was 0.244 ± 0.065. It can be observed that the standard deviation was higher for this paste than for the other tested pastes. The results indicated that total porosity values for fully carbonated cement pastes were similar for the different water-to-cement ratios studied herein. Although for younger pastes, a similar trend was reported in [53] for carbonated pastes with the same water to cement ratios as in this study. The authors reported increasing reductions of porosity after carbonation for increasing water-to-cement ratios in the HCP. A possible reason for this is that the initial carbonation rate of pastes with higher capillary porosity is higher. The decrease in porosity of cement pastes with w/c = 0.4 was in good agreement with the results obtained in [54,55] under the same accelerated carbonation conditions. The authors related such a large decrease in porosity to the attainment of carbonation equilibrium as shown in the mineralogic evolution.

It is worth noticing that the dimensions of the tested samples are not fully representative of the porosity of full-scale cementitious materials, i.e., entrained air and compaction voids are comparable or larger than those of the sample. Moreover, since an aqueous solution was used to measure porosity, swelling of the solid skeleton can lead to overestimation of the total porosity [42].

### 3.4. Relationship Between Total Porosity and Mechanical Properties

**Figure 10:**
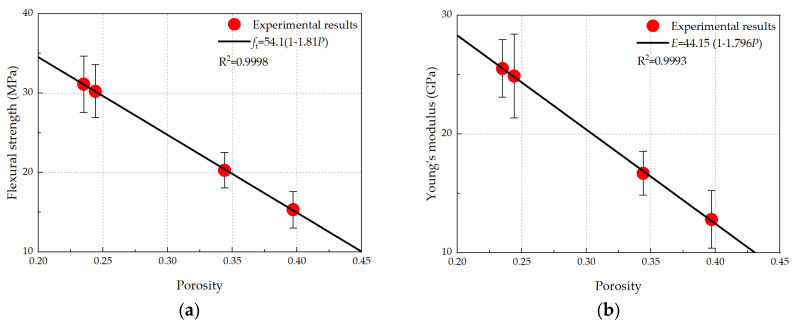
Relationship between porosity and mechanical properties: (**a**) flexural strength; (**b**) Young’s modulus.

The measured mean flexural strength and Young’s modulus are plotted with their corresponding porosities in Figure 10. Clearly, the mechanical properties decreased with the increase in porosity. A linear equation has been proposed to fit the porosity–mechanical properties and to show satisfactory results (R^2^ > 0.999). In terms of the porosity–Young’s modulus relationship, a similar trend was observed experimentally in recent studies on a model gypsum plaster material [56]. However, with respect to the porosity–strength relationship, an exponential relationship was observed in the model gypsum plaster material in which much larger size pores and porosity exist. It is worth mentioning that only four data-points were obtained in the current study; to further confirm the porosity–mechanical properties, more data-points over a larger range are required. Nevertheless, regardless of the w/c ratio and carbonation reaction, porosity appears to be the key factor dominating the strength and modulus properties of hydrated cement paste. This is in accordance with experimental observations reported in the literature [57,58,59].

## 4. Conclusions

In this work, micromechanical properties and porosity characterization of hydrated cement paste micro-beams before and after carbonation were performed. The small-scale test is promising to study the influence of carbonation on both total porosity and mechanical properties of HCP, as it can achieve a homogeneously carbonated specimen as well as shorten the carbonation treatment period. From the presented experimental work, the following conclusions can be drawn:

- After the accelerated carbonation regime followed in this study, micromechanical properties, i.e., flexural strength and Young’s modulus of HCP micro-beams, underwent a significant increase. Although noncarbonated HCP micro-beams with a w/c ratio of 0.4 feature much lower mechanical properties compared with a w/c ratio of 0.3, the carbonated HCP micro-beams showed similar mechanical properties for both w/c ratios (0.3 and 0.4).
- The micro-CT measurements revealed that the total porosity of ordinary HCP micro-beams was remarkably reduced after the accelerated carbonation treatment. The measured changes were in agreement with previous similar studies [53,54]. As the degree of hydration remained almost unchanged, the porosity reduction is attributed to the sole carbonation mechanism. More importantly, the total porosity of the studied carbonated HCP micro-beams decreased to a similar level regardless of the w/c ratio.
- The analysis of the relationship between porosity and micromechanical properties showed that regardless of the w/c ratio and carbonation process, the porosity appeared to be the main factor determining the mechanical properties of cement paste at the micro-scale. More specifically, both the flexural strength and elastic modulus increased with decreasing porosity.

We expect that the results presented in this study can be used as input to quantify and understand the effects of carbonation on the mechanical properties of hydrated ordinary Portland cement paste at larger scales, through numerical simulations. Such a study can be particularly useful to isolate the effect of carbonation-induced changes in porosity and minerology on the mechanical properties, from the influence of secondary degradation such as carbonation shrinkage and other scale-dependent degradation mechanisms (i.e., paste segregation, autogenous shrinkage).

## Figures and Tables

**Table 1:** Hydrates’ theoretical volumetric fractions (CH, CSH and AFm) for the studied HCP.

w/c	CH	CSH	AFm
0.3	0.175	0.288	0.162
0.4	0.177	0.291	0.163
